# Nutrients, Physical Activity, and Mitochondrial Dysfunction in the Setting of Metabolic Syndrome

**DOI:** 10.3390/nu15051217

**Published:** 2023-02-28

**Authors:** Gabriela de Oliveira Lemos, Raquel Susana Torrinhas, Dan Linetzky Waitzberg

**Affiliations:** Laboratory of Nutrition and Metabolic Surgery of the Digestive Tract (LIM 35), Department of Gastroenterology, University of São Paulo School of Medicine-FMUSP, Av. Dr. Arnaldo, 455, 2° andar, sala 2208, Cerqueira César, São Paulo 01246-903, SP, Brazil

**Keywords:** metabolic syndrome, mitochondrial function, sphingolipids, ceramides, diet, exercise

## Abstract

Metabolic syndrome (MetS) is a cluster of metabolic risk factors for diabetes, coronary heart disease, non-alcoholic fatty liver disease, and some tumors. It includes insulin resistance, visceral adiposity, hypertension, and dyslipidemia. MetS is primarily linked to lipotoxicity, with ectopic fat deposition from fat storage exhaustion, more than obesity per se. Excessive intake of long-chain saturated fatty acid and sugar closely relates to lipotoxicity and MetS through several pathways, including toll-like receptor 4 activation, peroxisome proliferator-activated receptor-gamma regulation (PPARγ), sphingolipids remodeling, and protein kinase C activation. These mechanisms prompt mitochondrial dysfunction, which plays a key role in disrupting the metabolism of fatty acids and proteins and in developing insulin resistance. By contrast, the intake of monounsaturated, polyunsaturated, and medium-chain saturated (low-dose) fatty acids, as well as plant-based proteins and whey protein, favors an improvement in sphingolipid composition and metabolic profile. Along with dietary modification, regular exercises including aerobic, resistance, or combined training can target sphingolipid metabolism and improve mitochondrial function and MetS components. This review aimed to summarize the main dietary and biochemical aspects related to the physiopathology of MetS and its implications for mitochondrial machinery while discussing the potential role of diet and exercise in counteracting this complex clustering of metabolic dysfunctions.

## 1. Introduction

Metabolic syndrome (MetS) is a constellation of major metabolic disorders, which include abdominal adiposity, insulin resistance, dyslipidemia, insulin resistance-induced hypertension, and inflammation [1,2]. Several healthcare organizations have established different criteria to define MetS, as detailed in Table 1 [1,2,3,4,5]. Although it is not included in major criteria for MetS diagnosis, non-alcoholic fatty liver disease (NAFLD) is closely related to MetS and is considered by some authors the hepatic representation of this syndrome. Additionally, it has been suggested that including NAFLD in MetS parameters enhances the screening of people with metabolic risk [6]. 

Regardless of the adopted definition, the presence of MetS is associated with increased risk for diabetes, cardiovascular disease, cancer, and non-alcoholic fatty liver disease, while its absence has been related to successful aging among community-dwelling elderlies [7]. The overall prevalence of MetS in the general population varies across countries and definitions (2.2–65.3%) but seems to be higher in women, urban residents, and older ages [8,9,10,11,12,13,14]. In individuals with obesity, the prevalence of MetS in Europe varied from 24% to 65% in women and from 43% to 75% in men [15].

The underlying cause of MetS has been a matter of debate, but experimental and human studies have included overnutrition and excess saturated fatty acids intake. At the molecular level, insulin resistance is the crucial cause of the MetS pathogenesis, apparently mediated by chronic systemic inflammation and oxidative stress involving mitochondrial dysfunction [16]. Indeed, ceramides, sphingolipids derived primarily from dietary long-chain saturated fatty acids (LCSFA), are believed to act as lipotoxic mediators of inflammation, mitochondrial and tissue dysfunction, and insulin resistance [17]. 

This review aimed to briefly describe the mitochondrial function and to summarize the main dietary and biochemical aspects related to MetS, its implications to mitochondrial machinery, and the potential role of diet modification and exercise in counteracting this complex metabolic cluster.

## 2. Mitochondrial Functioning

The major functions of mitochondria are energy and heat production. Acetyl-coenzyme A is the common metabolic intermediate that enters the tricarboxylic acid (TCA) cycle for adenosine triphosphate (ATP) synthesis. It may be generated from pyruvate as part of glucose catabolism or from fatty acids (FA) oxidation. Long-chain FA but not most of the medium-chain fatty acids (MCFA) require carnitine to shuttle into mitochondria [18]. 

Into the mitochondria inner space, electrons donated by NADH and FADH from the TCA cycle enter the electron transport chain, which consists of five enzyme complexes: NADH-CoQ reductase (complex I), succinate-CoQ reductase (complex II), CoQ-cytochrome c reductase (complex III), cytochrome c oxidase (complex IV), and ATP synthase (complex V). An electrochemical gradient is created by protons pumped into the intermembrane space—the mitochondria transmembrane potential (mΔψ). ATP is then generated from adenosine diphosphate and inorganic phosphate [18]. 

Conversely, proton leak from intermembrane space decreases mΔψ and favors heat production instead of ATP by uncoupling the TCA cycle and electron transport chain system. This process leads to the generation of reactive oxygen species (ROS) and is performed by specific proteins, or uncoupling proteins (UCPs) [19]. In ideal conditions, ROS are neutralized or scavenged by several enzymes, such as superoxide dismutase, glutathione peroxidase, peroxiredoxin III, and catalase. This process avoids mitochondrial dysfunction by preventing oxidative damage to the cell and mitochondrial proteins, desoxyribonucleic acid (DNA), and lipid membranes [19].

As part of the mitochondrial quality control, damaged mitochondria may enter the mitochondrial dynamics: (1) fission process (regulated by OPA1), (2) fusion process (regulated by Drp1 and Mfn1/2), and (3) mitophagy (PINK/Parkin pathway and autophagosome formation). However, extremely stressed conditions prompt mitochondrial permeability transition pore (mPTP) opening and mΔψ disrupting, leading to cytochrome c release, cytosolic caspases activation, and apoptosis [20]. Furthermore, mitochondrial biogenesis (increased mitochondrial cell content) is stimulated by peroxisome proliferator-activated receptor gamma coactivator-1α (PGC-1α) and allows for cell growth and proliferation [20].

Several nutrients (including vitamins and minerals) are precursors of mitochondrial enzymes and support mitochondrial function [18]. In this review, we focus on the role of macronutrients to contribute to or prevent MetS burden by affecting mitochondrial function. 

## 3. Dietary Macronutrients That Prompt MetS via Mitochondria

### 3.1. Unveiling Lipotoxicity from LCSFA 

There is a positive direct association between LCSFA consumption and MetS involving lipotoxicity that was previously explored in a systematic review [21]. Lipotoxicity is a term first coined by Young Lee and Roger H. Unger in 1994 to describe ẞ-cell abnormalities related to increased plasma levels of fatty acyl-CoA leading to insulin resistance. In their experiment, in obese prediabetic rodents, increased plasma free fatty acids (FFA) and triacylglycerol content in pancreatic islets preceded the loss of glucose-stimulated insulin secretion and correlated with glucose levels. These effects were alleviated by caloric restriction [22]. 

In obese conditions, increased plasma FFA is observed following increased LCSFA intake, as well as saturation of fat storage and loss of adipose tissue expandability [23]. Excess plasma FFA levels prompt a compensatory response to counteract lipid overload. This response includes: (1) triacylglycerol incorporation into fat cells for storage as “lipid droplets” (LD); (2) activation of oxidative programs in mitochondria and peroxisome; and (3) membrane lipid remodeling in cell membranes, with sphingolipids’ generation and functional membrane microdomains’ remodeling, or the “lipid rafts” [24]. 

Once fat storage is exhausted and the mitochondrial capacity of fatty acid oxidation is surpassed, the incomplete FA oxidation increases the generation of an intermediate metabolite (acylcarnitine), increases the lipid membrane remodeling through ceramide synthesis, and leads to an ectopic fat accumulation [24]. Several self-perpetuating mechanisms have been proposed to explain lipid-induced insulin resistance in MetS as listed in Table 2. 

Experimentally, excessive exogenous LCSFA intermediates the disruption of mitochondrial function, leading to mitochondrial damage and apoptosis. This effect can occur in different ways: mitochondrial membrane depolarization, mPTP opening, and ROS generation [35]. LCSFA are precursors of ceramides during de novo synthesis, which have been pointed out as central players in lipotoxic mitochondrial dysfunction (see [17]). Ceramides are key molecules of sphingolipid metabolism, and their generation involves several metabolic pathways, including sphingomyelin hydrolysis by sphingomyelinase and de novo synthesis by ceramide synthase (CerS)-isoforms 1 to 6 [36].

Ceramides interact with oxidized cytochrome c from mitochondria; decrease mitochondria transmembrane potential; increase ROS and oxidative stress; and initiate the mitochondrial outer membrane permeabilization with cytochrome c release, caspase activation, and apoptosis in a dose-dependent manner [37,38]. These events might be prevented by enhancing LCSFA oxidation through increased carnitine-palmitoyl-transferase 1 expression (which shuttles FA into mitochondria through ẞ-oxidation) or by glutathione activity [38,39].

CerS expression has different tissue distribution and acyl chain specificity. These properties seem to affect the effect of CerS. For instance, CerS6 activity favors insulin resistance and diet-induced steatohepatitis, while CerS2 activity (C22:0, C24:0, C24:1), mainly expressed in the liver, seems to protect from these conditions [40]. Thus, the ceramide composition also seems to have a functionally relevant aspect. In a prospective 7-year follow-up trial, baseline plasma levels of saturated fatty acid chain ceramides were positively associated with higher triacylglycerols levels, retroperitoneal and intraperitoneal fat masses, and homeostatic model assessment of insulin resistance (HOMA-IR), while they were negatively associated with high-density lipoprotein (HDL) cholesterol, adiponectin, and subcutaneous fat. Interestingly, unsaturated fatty acid ceramides had the opposite relation regarding visceral and subcutaneous fat and HOMA-IR [41]. 

Understanding the factors influencing CerS activity and the ceramide composition is relevant when considering that ceramides can impact health. For instance, in the PREDIMED study, a high ceramide concentration (a sum of C16:0, C22:0, C24:0, and C24:1 ceramides) at baseline was correlated to increased cardiovascular disease risk [42]. Similarly, baseline circulating Cer16, Cer18, Cer20, and Cer22 were associated to a higher diabetes risk [43]. In a cross-sectional analysis, individuals with type 2 diabetes (T2D) had higher Cer18:0, Cer20:0, Cer24:1, and total ceramides levels, where elevated Cer18:0 levels were inversely correlated with insulin sensitivity and directly correlated with circulating TNF-α levels [44]. 

Bariatric procedures are highly efficient for T2D therapy. In a recent study, patients with obesity and T2M who underwent Roux-en-Y gastric bypass who exhibited low serum ceramide levels at baseline, and those who presented ceramides decrease from the baseline to the second postoperative year, experienced persistent T2D remission 12 years after surgery. Using a linear mixed effect model ceramides inversely predicted T2D remission, independent of changes in body weight. These observations suggest a metabolic contribution of ceramide on insulin sensitization and T2D resolution independent of weight loss [45]. Indeed, increased ceramide transport in LDL also is found in T2D and does not correlate with obesity severity, but with insulin resistance. Moreover, the infusion of LDL ceramide in a mice model impaired insulin action and glucose homeostasis [46]. 

There is a special role for lipid oversupply and ceramide generation in metabolic disturbances. Increased plasma FFA and total muscle ceramides (primarily C18:1, C20:0, C22:0, C24:1, C24:0) are observed in individuals with obesity and T2D, as well as impaired muscle FFA oxidation in obese premenopausal women and individuals with T2D [47,48,49] Furthermore, there is an inverse relation between visceral adiposity and insulin-stimulated FFA uptake [49]. Curiously, ceramides do not seem to interfere with whole-body fat oxidation in an individual without T2D, whereas a persistent lipid oversupply results in excessive ceramide muscle accumulation in people with T2D [47].

Beyond lipotoxicity by sphingolipids, dietary quality and quantity of fat intake are associated with epigenetic regulation of energy and lipid metabolism through DNA methylation [50,51]. PGC1-α hypermethylation is associated with reduced gene expression and reduced mitochondrial DNA (mtDNA) content. These alterations are increased by exposing cells to free fatty acids [52,53]. The epigenetic modifications of liver mtDNA have been linked to insulin resistance and the severity of NAFLD [54,55]. MtDNA alterations precede mitochondrial dysfunction either with increased mtDNA content (not functional) or with decreased content and reduced oxidative phosphorylation [56]. Studies with NAFLD in mice show that a HFD is associated with a reduced half-life of mitochondrial proteins along with ATP deficiency [57].

Mitochondrial dysfunction in MetS is also supported by increased plasma levels of long acyl-carnitines (AcylCNs) and free carnitines (CNs) in patients with obesity and T2D. The formers are intermediate FA metabolites that play significant roles in cellular energy metabolism. Increased circulant levels of these molecules suggest incomplete ẞ oxidation of long-chain fatty acids. In T2D, increased medium-chain AcylCNs (C10- to C14) were associated with nuclear factor kappa B (NFkB) pathway activation. Thus, circulant AcylCNs and free CNs are helpful markers of mitochondrial and peroxisomal oxidation function [58,59]. 

**Figure 1:**
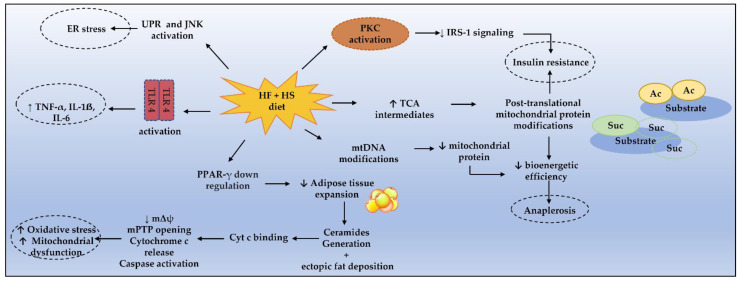
Mechanisms enrolling metabolic dysfunction in MetS. Cyt c, cytochrome c; ER, endoplasmic reticulum; HF, high fat; HS, high sugar; IRS-1, IL-1ẞ, interleukin 1ẞ; IL-6, interleukin 6; insulin receptor substrate 1; JNK, c-Jun N-terminal kinase; mPTP, mitochondrial permeability transition pore; mΔψ, mitochondrial transmembrane potential; PKC, protein kinase C; PPAR-γ, peroxisome proliferator-activated receptor γ; TLR4, toll-like receptor 4; UPR, unfolded protein response, TNF-α, tumor necrosis factor α.

Altogether, these findings support the central role of long-chain ceramides’ generation from dietary LCSFA in lipotoxicity, which along with the mechanisms illustrated in Figure 1, prompts mitochondrial dysfunction and MetS pathogenesis.

### 3.2. Protein Modifications from Fructose- and Sugar-Sweetened Foods

Beyond a high-fat diet (HFD), a high-sugar diet also is associated with MetS [60]. Hepatic epigenetic and post-translational mitochondrial proteins’ modifications (PTM) have been described following long-term sugar intake, including mitochondrial DNA hypomethylation with protein hyperacetylation and/or hypo-succinylation (when combined with HFD). These modifications can rise from an accumulation of acetyl-coenzyme A, as an intermediate of the TCA, leading to disrupted glucose, lipid, and protein metabolism [31,61]. 

Fructose metabolism induces the activation of adenosine monophosphate deaminase, leading to uric acid generation and mitochondrial oxidative stress through distinct pathways. These include the activation of nicotinamide adenine dinucleotide phosphate oxidase subunit NOX4, aconitase inhibition (TCA cycle enzyme), and citrate overload [62]. L6 myotubes exposed to high fructose concentration were shown to induce mitochondrial dysfunction due to reduced mitochondrial enzyme activity, decreased mitochondrial membrane potential and mitochondrial electron transport chain, and disrupted energy metabolism. In turn, these events lead to increased ROS, reactive nitrogen species, and apoptosis [63]. 

It is suggested that the impact of sugars on MetS risk may be related to its caloric component. A United States survey with teenagers correlated increased sugar ingestion with progressive MetS risk, despite body mass index (BMI) values, physical activity, and total energy intake, mainly when consumed above 70 g/day [64]. Conversely, a meta-analysis showed an association of fructose consumption to MetS only when this was consumed as extra energy in hypercaloric diets (>+21% to 35% extra energy) [65]. In a large cohort study, the total carbohydrate intake showed an association with mortality where the lower mortality risk ranged between 50 and 55% of total energy intake [66]. 

The dietary source of sugar is an important issue concerning MetS risk, with sugar-sweetened beverages (SSB) conferring a higher risk and yogurt and fruits conferring a lower risk [67]. Together with foods with added sugar, SSB consumption is often a source of high sugar/fructose intake in children, adolescents, and adults and has been linked to insulin resistance and MetS [64,67]. In the Framingham Offspring Cohort, the frequency of SSB consumption correlated to plasma Cer16:0, Cer22:0, and Cer24:0. In individuals with prediabetes or T2D, plasma Cer24:0 correlated to more recurrent SSB ingestion [68]. In addition, a meta-analysis showed that subjects consuming more than 1–2 serving of SSB/day exhibited a 26% and 20% higher risk of developing T2D and MetS, respectively [69]. Curiously, artificially sweetened beverages (diet or non-carbohydrate low calorie foods) have shown a linear dose–response relationship in MetS risk [70]. 

In the KNHANES survey (2007–2014), a higher carbohydrate intake (≥ 74.2% of energy intake) correlated to MetS risk in women irrespective of dietary lipid composition [71]. Nevertheless, the long-term association of high fat + high sugar diet (HFSD) seems to be the most deleterious combination, exacerbating every isolated nutrient overload toxicity and culminating in mitochondrial inefficiency, reduced fatty acid utilization, and tissue lipid overload. Indeed, the HFSD diet has been used as an effective experimental model to induce MetS in rats [72]. 

Overall, a high-sugar diet prompts hepatic epigenetics and PTM, which are related to impaired glucose, lipid, and protein metabolism along with mitochondrial dysfunction. Therefore, sugar intake should be discouraged in individuals aiming to maintain a healthy status or to manage MetS. Artificially sweetened beverages (with no sugar) also have shown to be positively associated to MetS, but the mechanisms enrolled remain poorly elucidated. 

## 4. Dietary Macronutrients That Can Protect from MetS via Mitochondria

### 4.1. Long-Chain Unsaturated Fatty Acids 

#### 4.1.1. Polyunsaturated Fatty Acids (PUFAs)

Intake of N-3 PUFAs, but not n-6 PUFAs, has been associated with a 26% lower MetS risk in a large systematic review and meta-analysis, primarily in Asian populations [53]. N-3 PUFAs, primarily eicosapentaenoic acid (EPA), are precursors of less pro-inflammatory eicosanoids (3-series prostaglandins and thromboxane and 5-series leukotrienes) than those produced from the n-6 PUFAs, like arachidonic acid, and are also precursors of specialized pro-resolving mediators (SPMs)—resolvins (EPA and DHA), protectins, and maresins (DHA). When incorporated into cell membranes, EPA and DHA increase membrane fluidity and enhance insulin signal transduction [33,73]. They also are naturally PPARs ligands, able to inhibit NFκB signaling and exert a role in glucose and lipid metabolism, as well as in inflammation relief [74]. 

In the genetically modified mice model, endogen recovery of n-3 PUFAs reverted inflammation and prevented insulin resistance and obesity-induced inflammation [75]. N-3 PUFAs downregulate Sptlc3 and Degs2-CerS corresponding genes, reducing CerS activity (Cer16:0, Cer18:0, Cer20:0, Cer22:0, and Cer24:0) and decreasing hepatic steatosis in a hyperhomocysteinemia-induced hepatic steatosis mouse model [76]. This lipidomic improvement finding was previously described in a pre-diabetic mice model supplemented with fish oil (rich in EPA and DHA) [77]. 

The effect of n-3 PUFAs on CerS seems to be fat tissue-dependent. In mice, it reverses the experimentally induced increase in CerS2-6 activity at perigonadal (visceral fat) but not at subcutaneous fat [78]. In the MetS frame, this observation suggests that n-3 PUFAs impact visceral lipid infiltration more than the loss of adipose tissue. Indeed, in muscle cells cultured with palmitate, the addition of EPA, DHA, and alpha-linoleic acid decreased cell diglycerides and ceramides’ content as well as improved glucose uptake. In addition, only EPA and DHA prevented palmitate-impaired AKT phosphorylation, increased palmitate oxidation, decreased its incorporation in DG, and decreased protein kinase Cθ activation [79]. 

Systematic reviews and meta-analyses have found that fish-oil/n-3 PUFAs supplementation in patients with metabolic disturbances and T2D is associated with improvement in cardiometabolic profile. It has been shown to enhance glucose metabolism (increased insulin sensitivity and decreased fasting plasma glucose and glycated hemoglobin) and lipid profile (lowered total and low-density lipoprotein cholesterol and TG and increased high-density lipoprotein cholesterol). Additionally, it was shown to decrease inflammatory biomarkers (TNF-α and C-reactive protein) and body weight [80,81]. 

The effect of n-3 PUFAs may depend on its amount and the disease progression. In subjects at high risk of cardiovascular disease, fatty fish intake decreased several plasmatic lipid species, including ceramides, when compared to the lean fish intake group and subjects at low risk of cardiovascular disease [33]. Moreover, in T2D subjects, the enhanced insulin signaling effect seems to be associated with a low dietary n-6 to n-3 PUFAs ratio [82]. 

Indeed, a low n-6 to n-3 PUFAs ratio seems to be associated with overall MetS improvement. Experimentally, the mechanism associated includes an enhanced mitochondrial function via inhibition of the mammalian target of rapamycin complex 1 (mTORC1) signaling and upregulation of the mitochondrial electron transport chain and tricarboxylic acid cycle pathways [83]. In addition, n-3 PUFAs improve mitochondrial ATP synthesis and decrease ROS generation via increased mitofusin 2 (Mfn2) expression [84]. 

Although n-3 PUFAs are highly susceptible to lipid peroxidation, EPA and DHA have shown antioxidant effects, including the regulation of the nuclear factor erythroid-2 related factor 2 (Nrf2) activity, responsible for the transcription of the main antioxidant enzymes. In T2D patients, the oral supplementation of n-3 PUFAs (2.7 g for 10 weeks) versus a control group was associated to increased Nrf2 expression and antioxidant capacity on lipid peroxidation markers [85]. It is also has been shown that n-3 PUFAs intake decreases the amount of urinary F2-isoprostanes, a group of stable products from lipid peroxidation [86]. 

A systematic review showed that n-3 PUFAs supplementation increased total antioxidant capacity and glutathione peroxidase activity and decreased malondialdehyde [87]. In a randomized, double-blinded study, in patients with non-alcoholic steatohepatitis (NASH), the n-3 PUFAs supplementation changed the proteomics profile favoring proteins related to enhanced antioxidant capacity along with intracellular lipid transportation, oxidative phosphorylation, and decreased endoplasmic reticulum stress [88]. 

As summarized above, n-3 PUFAs have several effects on glucose and lipid metabolism and gene expression by mitigating lipotoxicity, inflammation, and oxidative stress biomarkers that are closely related to mitochondrial function. The consumption of n-3 PUFAs also shows an improvement in MetS features. These alterations are related to the epigenetic effects of this family of fatty acids and their individual biophysical and biochemical properties. Thus, their consumption either as food or supplement may be a good strategy to improve MetS.

#### 4.1.2. Monounsaturated Fatty Acids (MUFAs)

MUFAs are largely abundant in olive oil (OO) and exert epigenetic effects on ẞ-oxidation and triglyceride (TG) synthesis in muscle cells via PPAR-α and protein kinase A (PKA) signaling [89]. The formers are nuclear receptors that regulate lipid and bile acid metabolism, and the other is a key intracellular enzyme in the regulation of energy metabolism, including in mitochondria. Both these molecules have shown to revert palmitate-induced insulin resistance and inflammation [90,91].

MUFA consumption is associated with improvements in MetS risk factors either alone or associated with a Mediterranean diet (MedDiet) or PUFAs [92]. In the PREDIMED study, when compared to a low-fat diet, a MedDiet supplemented with OO or nuts (also rich in MUFAs) decreased body weight and enhanced glucose metabolism [93]. In the same cohort, OO or nut supplementation plus a MedDiet reduced the risk for cardiovascular disease in individuals with high ceramide levels at baseline [42]. The mechanisms involved in these benefits seem to include an augment of the antioxidant potential by increasing superoxide dismutase and catalase levels and decreasing xanthine oxidase [94]. OO also showed a synergic effect on fish oil in improving lipid profile and decreasing peroxidation biomarkers [95]. Indeed, compared to other therapies specially designed to manage MetS (but not placebo), MUFA administration showed a similar benefit on MetS parameters and antioxidant capacity [96]. 

The improvement in MetS parameters by OO supplementation may be followed by improvement in other related clinical conditions. The extra virgin OO supplementation decreased the fatty liver index (FLI), alanine transaminase, and inflammatory markers (IL-6, LI-17, TNF-, IL-1B) [97]. In the PREDIMED-Reus study, without calorie restriction, a decreased occurrence of T2D was observed in the group fed OO plus a MedDiet, compared to MedDiet alone and a control diet [98]. This finding was corroborated by a systematic review where the risk of T2D was decreased by 13% in individuals consuming ~15–20 g OO per day. In this review, previously diagnosed subjects with T2D, the OO ingestion was associated to lower HbA1c and FPG levels [99]. 

Taken together, these observations suggest that MUFAs enhance mitochondrial oxidation, increase antioxidant capacity, decrease inflammatory and peroxidation biomarkers, and improve MetS parameters; thus, it may be an easy dietary approach to manage this syndrome.

### 4.2. Medium-Chain Saturated Fatty Acids (MCFAs)

The physical-chemical characteristics of fatty acids interfere with metabolism. Compared to LCSFAs, MCFAs are more efficiently oxidized into mitochondria [100]. MCFAs have facilitated absorption, transport, and metabolism due to their direct entry to the portal vein from the intestinal lumen, albumin-binding transportation, and prompt mitochondrial ẞ-oxidation, partially dismissing carnitine transportation [101]. These MCFAs’ properties seem to favor important mitochondrial functions to avoid MetS.

In insulin-resistant THP-1 macrophage cultures, the incubation with MCFAs (lauric acid) improved mitochondrial content and biogenesis, restored mΔψ and ATP production, decreased ROS generation, increased peroxisome proliferator-activated receptor (PPAR)-γ expression along with its coactivator 1-alpha (PGC-1α), and increased the gene expression of mitochondrial transcription factor A (which has a role in mitochondrial biogenesis regulation) in a dose-dependent manner [102]. MCFAs also have been shown to stimulate uncoupling protein-1 expression even under caloric restriction and to play a key role in thermogenesis and body weight maintenance [103,104]. Indeed, MCFAs supplementation is associated with increased thermogenesis and energy expenditure [105,106]. A meta-analysis comparing to LCSFAs supported an effect of MCFAs on the improvement in body composition, reduction of waist circumference, visceral fat, and weight loss [107]. 

Mitochondrial dysfunction can promote liver inflammation and its progression to liver disease, a common feature of the MetS [108]. In an HFD-fed mice model, a dose-dependent isocaloric substitution of dietary lipids by MCFAs resulted in PPAR-α activation, increased ẞ- and ω-oxidation, enhanced mitochondrial respiration, and decreased lipid content and hepatic steatosis [109]. Another study found divergent findings with a worsening in metabolic profile and hepatic lipid content. In this experiment, MCFAs were added to HFD instead of replacing the lipid fraction of HFD [110]. 

In humans, MCFAs are shown to increase HDL and apolipoprotein A-1 compared to LCSFA [111]. In addition, MCFAs’ consumption is associated with decreased low-density lipoprotein cholesterol (LDL-C) compared to animal oils, but increased LDL-C compared to plant oils [112]. Considering that liver damage has shown an association with total cholesterol, HDL, and LDL levels, MCFAs may protect from liver damage in humans [113]. 

MCFAs are less prompt to lipid peroxidation than unsaturated FAs, and are not precursors of inflammatory mediators, suggesting a low oxidative potential. In a mice model, the supplementation of virgin coconut oil (rich in MCFAs) showed to increase the total antioxidant activity, individual superoxide dismutase, catalase, and glutathione peroxidase activities and decreased malondialdehyde and lactate dehydrogenase levels [114,115]. This antioxidant potential capacity has been correlated to enhanced insulin sensitivity and hepatic steatosis improvement [115,116]. Conversely, increased myocardial oxidative damage after MCFA supplementation was observed in a mice model, but this finding did not translate to human studies [117].

In addition to reports on the positive effects of MCFAs on MetS features, a randomized controlled trial found an association of increased endothelial dysfunction markers with virgin coconut oil ingestion, suggesting caution upon its supplementation [118]. Moreover, although MCFAs have a comparable effect to olive oil (OO) in reverting MetS parameters (fasting serum glucose, total cholesterol, and diastolic blood pressure) [119], there are conflicting findings regarding hepatic lipid content and steatosis improvement [120]. 

In summary, an isocaloric dietary lipid substitution including low MCFAs amounts may exert beneficial effects on MetS features due to enhanced mitochondrial function and biogenesis, thermogenesis regulation, and increased lipid oxidation.

### 4.3. Protein Supplements 

Increased plasma-free amino acids (PFAAs) and branched-chain amino acids (BCAAs; isoleucine (Ile), leucine (Leu), and valine (Val)) have been described in individuals with T2D, hypertension, dyslipidemia, and MetS from different ethnicities (Asian, Mediterranean, Caucasian, and Afro-American). The type of amino acid may be important for this association with MetS and its metabolic components [121,122,123]. In a Japanese Cohort, tyrosine, alanine, and BCAA positively correlated with metabolic profile (impaired glucose metabolism and abdominal adiposity), while glycine presented a negative correlation with these features. Nevertheless, the total PFAA index has been correlated to abdominal adiposity and increased risk for developing MetS, even after adjusting for other common risk factors (age, gender, waist circumference, BMI, glucose, and lipid profile) [124]. 

Increased levels of BCAA are associated with NAFLD, independent of gender, insulin resistance, and obesity. It seems to be related to impaired BCAA catabolism in adipose tissue, which primarily supports the adipose tissue dysfunction in MetS [125]. In individuals with NAFLD, decreased levels of BCAA also are associated with liver mitochondrial damage and increased levels of metabolites from Krebs cycle [126]. A 50% increase in reactions for reestablishing TCA intermediates—the so-called anaplerotic fluxes—was observed in humans and mice models with NAFLD [127,128]. These alterations were shown to positively correlate with the increased liver synthesis of aminotransferases and consequently increased gluconeogenesis, which could explain the increased amino acid levels in MetS [129].

Along with increased plasma BCAA, an increased short acylcarnitine to all carnitine ratio was observed in individuals with obesity, metabolic unwellness, and T2D [130]. Short acyl chain carnitines may derive from ketone bodies, BCAA metabolism, or even glucose to counteract the excess of TCA in conditions of energetic oversupply or to reestablish energy substrate in fasting conditions. Both situations are characterized by increased mitochondrial lipid fluxes and oxidation [131,132]. 

Similarly, increased short acylcarnitines (C3 and C5) derived from BCAA metabolism correlate with plasma BCAA levels, but not with BCAA intake, in individuals with obesity and MetS [133]. Decreased hydroxydecanoyl carnitine and methylglutarylcarnitine (medium-chain acylcarnitines, which are intermediates of FA oxidation) are also observed in subjects with MetS, which could also depend on increased fatty acids fluxes and utilization [134]. Indeed, plasmatic glutamic (amino acid metabolism) and lactic (anaerobic energy production) acids were positively associated to MetS parameters (obesity and lipids and glucose-impaired metabolism). In parallel, glutamic acid and 2-ketoglutaric acid (amino acid metabolism and urea cycle) also showed a positive significant association with the levels of aspartate transaminase and alanine transaminase. These enzymes are primarily located in the liver, which catalyzes amino acid transamination, especially glutamate [135]. Taken together, the observations above suggest a reduced oxidative phosphorylation and disrupted amino acid metabolism.

One can then propose that impaired mitochondrial function may underlie protein catabolism in MetS due to the anaplerotic protein/amino acids metabolism [31]. An important point concerning this topic was described by two different studies reporting PFAA modifications when individuals with MetS were submitted to lifestyle modification and weight loss and experienced PFAA normalization [136,137]. In another study, increased BCAA intake correlated to MetS in subjects with ≥7% weight gain, but not in individuals with <7% weight gain for 8.9 years follow-up [138]. 

Therefore, increased PFAA in MetS seems not to be a direct causative relationship but rather a consequence of deficient energy generation from lipids and glucose, mainly due to mitochondrial dysfunction and insulin resistance. Corroborating this hypothesis, two different systematic reviews with up to 2344 subjects demonstrated an improvement of different MetS features following a BCAA-containing whey protein supplementation, including body composition, blood pressure, and glucose and lipid metabolism [139,140]. Another systematic review suggests that soy protein intake with isoflavones may confer enhanced cardiovascular protection over animal-based proteins given its increased total cholesterol, LDL, and blood pressure-lowering properties [141].

Altogether, these findings suggest that the increased BCAA levels found in individuals with MetS and T2D seem to be related to an anaplerotic mechanism due to impaired mitochondrial function that is improved by weight loss. Additionally, a plant-based protein diet along with whey protein supplementation has been shown to improve MetS parameters.

## 5. The Role of Exercise in MetS

Exercise training has a well-described cardiovascular benefit and has been shown to improve MetS biomarkers by reducing body weight, waist circumference, and blood pressure, as well as improving circulating lipid and glucose profiles [142]. The proposed mechanism includes adaptative metabolic modifications in response to increased metabolic tissue demands and increased generation of ROS and reactive nitrogen species [143]. Experimentally, mice submitted to endurance training have shown improvements in antioxidant capacity and lipid peroxidation markers, enhanced bioenergetics (increased oxygen consumption and increased mitochondrial complexes), enhanced mitochondrial biogenesis and dynamics (increased expression of PGC-1 α and Mfn1 protein, fission inhibition, and fusion induction), and mitophagy regulation [144,145,146].

Indeed, physical exercise has been shown to upregulate PPARγ/PGC-1 α activity, which is associated with improvement in lipid and anti-inflammatory profile [147,148]. It also exerts epigenetic modifications by reducing PGC-1 α methylation, favoring mitochondrial biogenesis [149]. Therefore, physical activity may improve the MetS by enhancing mitochondrial function. In patients with T2D, endurance training improved mitochondrial respiration and increased maximal oxygen uptake (VO2max) when combined or not with resistance training [150,151]. Along with cardiorespiratory fitness, aerobic exercise is associated with a reduction in liver fat and enzymes in individuals with MetS [152]. In healthy men and post-menopausal women, endurance and resistance training have shown to increase mitochondrial O2 respiration coupled to ATP synthesis, which is a marker of mitochondrial function [153,154]. 

These observations above point out an improvement in the mitochondrial oxidation capacity after exercise. Linked to enhanced energetic efficiency, endurance training in individuals with obesity and T2D has also been shown to decrease diacylglycerol, total ceramides, and Cer14:0 content in skeletal muscle with a positive correlation with insulin sensitivity [155,156]. These microenvironment adaptations in response to physical exercise contrast to the ceramide skeletal muscle content (C16:0 and C20:0) found in aging that is positively correlated with NFkB signaling activation and impaired anabolic signaling (Akt, FOXO1, and S6K1 molecules) [157]. This may explain the anabolic resistance found in the elderly. These also may reflect on inflammation markers and clinical outcomes. A systematic review showed a decrease in inflammatory markers (TNF-α, CRP, IL-8) and an increase in IL-10 in subjects with MetS submitted to either regular aerobic or combined exercises [158].

The metabolic response to physical exercise may be impacted by the kind of training program applied. A meta-analysis evaluated different exercise modalities compared to a non-exercising group (≥3 days/week during ≥12 weeks) on MetS parameters. They found a significant decrease in WC, fasting glucose, diastolic blood pressure, and TG and an increase in HDL and cardiorespiratory fitness (+4.2 mL/kg/min). Relevant benefits of resistance exercise training (RET) over aerobic exercise training (AET) were not observed on MetS features [159]. However, another meta-analysis that specifically addressed comparing aerobic, resistance, and combined exercising training (CET) found CET to have a greater impact on glucose metabolism and TG, while RET was more effective in reducing body fat and AET in reducing BMI [160]. Moreover, the exercise intensity may also impact its metabolic response. The high-intensity aerobic exercise showed to have a greater impact on VO2max gain and systolic blood pressure decrease than moderate intensity [142]. 

**Figure 2:**
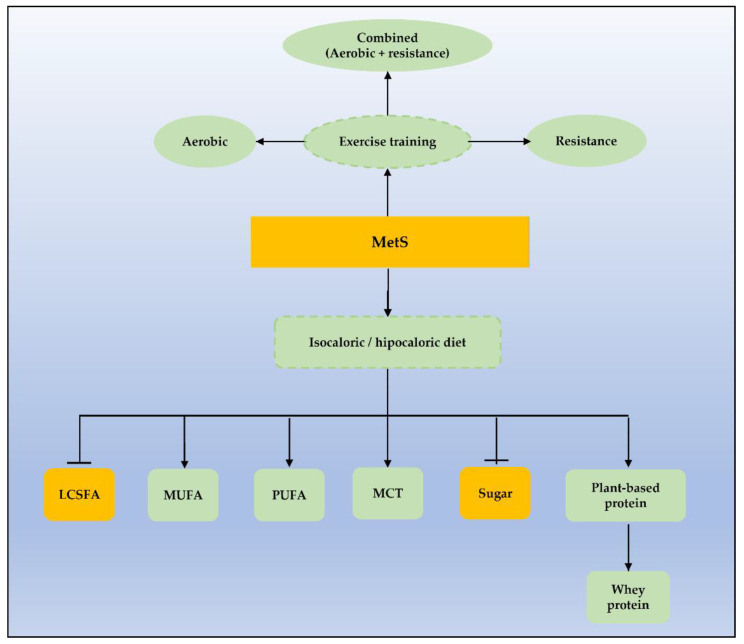
Dietary and exercise approaches targeting MetS. LCSFA, long-chain saturated fatty acids; MCT, medium-chain fatty acids; MUFAs, monounsaturated fatty acids, PUFAs, polyunsaturated fatty acids.

When associated with diet intervention, physical activity is more effective in reverting MetS, compared to exercise alone or diet alone (67.4%, 23.5%, and 35.3% respectively) [161]. The impact of a structured combined exercise program on weight loss is suggested to increase with long-term interventions [162]. Indeed, high cardiovascular fitness aligned with a healthy dietary pattern is described to have an inverse association with MetS risk [163], as seen in Figure 2.

Briefly, it seems that exercise has a pleiotropic effect on nutrients, metabolism, mitochondrial function, and biogenesis that prompts cardiovascular fitness and improvement of inflammation markers and MetS features. Indeed, the benefits of exercise are enhanced when combining aerobic and resistance training.

## 6. Conclusions

Overnutrition and a high-fat/high-sugar diet have been shown to be associated to MetS in several animal models and epidemiological trials by impairing fat storage capacity and prompting ectopic fat accumulation. A high-fat/high-sugar diet leads to increased ceramide generation and mitochondrial post-translational protein modification, which play a central role in mitochondrial dysfunction, insulin resistance, epigenetic modification, and MetS development. Dietary interventions to improve clinical outcomes in MetS should include the intake of a low calorie or isocaloric diet, enriched with MUFAs, PUFAs, fruits and yogurt with low sugar content, and low sugar-sweetened beverage and artificially sweetened beverage intake. Intake of plant-based protein, eventually supplemented with whey protein, is recommended, as well as the inclusion of low-dose medium-chain fatty acids intake. When combined with structured exercise programs, irrespective of exercise modality, dietary modifications may mitigate MetS biomarkers and improve weight loss with time-dependent effectiveness.

## Figures and Tables

**Table 1:** Available definitions for metabolic syndrome considering common metabolic disturbances.

Feature	Definition				
	WHO ^1^	NCEP-ATP III ^2^	IDF ^3^	AHA-NHLBI ⁴	JIS ⁵
Impaired glucose metabolism	DM: FG ≥ 126 or 2 h PGL ≥ 200 mg/dL or IGT: FG < 126 and 200 < 2 h PGL ≥ 140 mg/dL or IFG: FG ≥ 110 and 126 < 2 h PGL< 140 mg/dL	FG ≥ 110 mg/dL	FG ≥ 100 mg/dL Or previously diagnosed DM	FG ≥ 100 mg/dL Or current drug treatment for ↑ glucose	FG ≥ 100 mg/dL Or current drug treatment for ↑ glucose
Central Obesity	WHR > 0.9 M and >0.85 W or BMI > 30 Kg/m^2^	WC > 102 cm M WC > 88 M	WC > 102 cm M WC > 88 M	WC ≥ 102 cm M WC ≥ 88 M	Specific definitions by country and population
HDL	<35 mg/dL M <39 mg/dL W	<40 mg/dL M <50 mg/dL W	<40 mg/dL M <50 mg/dL W	<40 mg/dL M <50 mg/dL W or current drug treatment for ↓ HDL	<40 mg/dL M <50 mg/dL W
Triglyceride	≥150 mg/dL	≥150 mg/dL	≥150 mg/dL or previously diagnosed lipid abnormality	≥150 mg/dL or current drug treatment for ↑ triglyceride	≥150 mg/dL
Blood Pressure	≥160/90 mmHg	≥130/≥85 mmHg	≥130/≥85 mmHg Or treatment of previous hypertension	≥130/≥85 mmHg Or current antihypertensive treatment	≥130/≥85 mmHg Or current antihypertensive treatment
Microalbuminuria	≥20 µg/min or albumin:creatinine ratio ≥20 mg/g	--	--	--	--
**Criteria**	Glucose-impaired metabolism + 2 other features	≥3 features	Central obesity + 2 other features	≥3 features	≥3 features

Diagnostic criteria for MS, according to: ^1^-the World Health Organization; ^2^-the Third Report of the National Cholesterol Education Program, the Expert Panel on Detection, Evaluation, and Treatment of High Blood Cholesterol in Adults—Adult Treatment Panel III; ^3^-the International Diabetes Federation; ^4^-the American Heart Association, and the National Heart, Lung, and Blood Institute; and ^5^-the Joint Interim Statement of the International Diabetes Federation Task Force on Epidemiology and Prevention; National Heart, Lung, and Blood Institute; American Heart Association; World Heart Federation; International Atherosclerosis Society; and International Association for the Study of Obesity. BMI: body mass index, DM: diabetes mellitus; IGT: impaired glucose tolerance; IFG: impaired fasting glycaemia; FG: fasting glucose; PGL: post glucose load; WHR: waist-to-hip ratio; WC: waist circumference; M: men; W: women; ↑: increased; ↓: decreased.

**Table 2:** Mechanisms proposed to explain insulin resistance induced by lipids.

Causes	Consequences	References
TLR4 activation in adipocytes, macrophages, and skeletal cells	Pro-inflammatory cytokine production (TNF-α, IL-1ẞ, IL-6)	[25]
PKC activation	↓ insulin-stimulated IRS-1 tyrosine phosphorylation	[26,27,28]
Mitochondrial dysfunction	↑ oxidative stress and anaplerosis	[29,30,31]
↓ n3-PUFAs intake	↑ pro-inflammatory cytokine production and impaired insulin signaling	[32,33]
UPR and JNK activation	ER stress	[30,34]

Causes and consequences of lipid-induced insulin resistance in MetS: ER, endoplasmic reticulum; IRS-1, IL-1ẞ, interleukin 1ẞ; IL-6, interleukin 6; insulin receptor substrate 1; JNK, c-Jun N-terminal Kinase; n-3 PUFAs, n-3 polyunsaturated fatty acids; PKC, protein kinase C; UPR, unfolded protein response, TNF-α, tumor necrosis factor α; TLR4, toll-like receptor 4; ↑, increased; ↓, decreased.

## Data Availability

Not applicable.

## References

[1] International Diabetes Federation (2006). The IDF Consensus Worldwide Definition of the METABOLIC SYNDROME. Int. Diabetes Fed..

[2] Alberti K.G.M.M., Zimmet P.Z. (1998). Definition, Diagnosis and Classification of Diabetes Mellitus and Its Complications Part 1: Diagnosis and Classification of Diabetes Mellitus Provisional Report of a WHO Consultation ORIGINAL ARTICLES. Diabet. Med..

[3] Expert Panel on Detection, Evaluation, and Treatment of High Blood Cholesterol in Adults (2001). Executive Summary of the Third Report of the National Cholesterol Education Program (NCEP) Expert Panel on Detection, Evaluation, and Treatment of High Blood Cholesterol in Adults (Adult Treatment Panel III). JAMA J. Am. Med. Assoc..

[4] Grundy S.M., Cleeman J.I., Daniels S.R., Donato K.A., Eckel R.H., Franklin B.A., Gordon D.J., Krauss R.M., Savage P.J., Smith S.C. (2005). Diagnosis and Management of the Metabolic Syndrome: An American Heart Association/National Heart, Lung, and Blood Institute Scientific Statement. Circulation.

[5] Alberti K.G., Eckel R.H., Grundy S.M., Zimmet P.Z., Cleeman J.I., Donato K.A., Fruchart J.C., James W.P., Loria C.M., Smith S.C. (2009). Harmonizing the metabolic syndrome: A joint interim statement of the International Diabetes Federation Task Force on Epidemiology and Prevention; National Heart, Lung, and Blood Institute; American Heart Association; World Heart Federation; International Atherosclerosis Society; and International Association for the Study of Obesity. Circulation.

[6] Fu C.E., Yong J.N., Ng C.H., Nah B., Chew N.W.S., Chin Y.H., Kong G., Tan D.J.H., Lim W.H., Lim L.K.E. (2023). Original article: The prognostic value of including non-alcoholic fatty liver disease in the definition of metabolic syndrome. Aliment. Pharmacol Ther..

[7] Lin Y.H., Chiou J.M., Chen T.F., Lai L.C., Chen J.H., Chen Y.C. (2021). The Association between Metabolic Syndrome and Successful Aging- Using an Extended Definition of Successful Aging. PLoS ONE.

[8] Scuteri A., Laurent S., Cucca F., Cockcroft J., Cunha P.G., Mañas L.R., Mattace Raso F.U., Muiesan M.L., Ryliškytė L., Rietzschel E. (2015). Metabolic Syndrome and Arteries Research (MARE) Consortium. Metabolic syndrome across Europe: Different clusters of risk factors. Eur. J. Prev. Cardiol..

[9] de Siqueira Valadares L.T., de Souza L.S.B., Salgado Júnior V.A., de Freitas Bonomo L., de Macedo L.R., Silva M. (2022). Prevalence of Metabolic Syndrome in Brazilian Adults in the Last 10 Years: A Systematic Review and Meta-Analysis. BMC Public Health.

[10] Ansarimoghaddam A., Adineh H.A., Zareban I., Iranpour S., HosseinZadeh A., Kh F. (2018). Prevalence of Metabolic Syndrome in Middle-East Countries: Meta-Analysis of Cross-Sectional Studies. Diabetes Metab. Syndr. Clin. Res. Rev..

[11] Gutiérrez-Solis A.L., Datta Banik S., Méndez-González R.M. (2018). Prevalence of Metabolic Syndrome in Mexico: A Systematic Review and Meta-Analysis. Metab. Syndr. Relat. Disord..

[12] Ranasinghe P., Mathangasinghe Y., Jayawardena R., Hills A.P., Misra A. (2017). Prevalence and Trends of Metabolic Syndrome among Adults in the Asia-Pacific Region: A Systematic Review. BMC Public Health.

[13] Oguoma V.M., Nwose E.U., Richards R.S. (2015). Prevalence of Cardio-Metabolic Syndrome in Nigeria: A Systematic Review. Public Health.

[14] Hirode G., Wong R.J. (2020). Trends in the Prevalence of Metabolic Syndrome in the United States, 2011–2016. JAMA.

[15] van Vliet-Ostaptchouk J.V., Nuotio M.L., Slagter S.N., Doiron D., Fischer K., Foco L., Gaye A., Gögele M., Heier M., Hiekkalinna T. (2014). The prevalence of metabolic syndrome and metabolically healthy obesity in Europe: A collaborative analysis of ten large cohort studies. BMC Endocr. Disord..

[16] Vona R., Gambardella L., Cittadini C., Straface E., Pietraforte D. (2019). Biomarkers of Oxidative Stress in Metabolic Syndrome and Associated Diseases. Oxid Med. Cell. Longev..

[17] Chaurasia B., Summers S.A. (2020). Ceramides in Metabolism: Key Lipotoxic Players. Annu. Rev. Physiol..

[18] Pieczenik S.R., Neustadt J. (2007). Mitochondrial Dysfunction and Molecular Pathways of Disease. Exp. Mol. Pathol..

[19] Jia G., Aroor A.R., Sowers J.R. (2014). Estrogen and Mitochondria Function in Cardiorenal Metabolic Syndrome. Prog. Mol. Biol. Transl. Sci..

[20] Li J., Li J., Chen Y., Hu W., Gong X., Qiu H., Chen H., Xin Y., Li H. (2022). The Role of Mitochondria in Metabolic Syndrome-Associated Cardiomyopathy. Oxid. Med. Cell. Longev..

[21] Julibert A., Del Mar Bibiloni M., Tur J.A. (2019). Dietary Fat Intake and Metabolic Syndrome in Adults: A Systematic Review. Nutr. Metab. Cardiovasc. Dis..

[22] Lee Y., Hirose H., Ohneda M., Johnson J.H., Mcgarry J.D., Unger R.H. (1994). Beta-Cell Lipotoxicity in the Pathogenesis of Non-Insulin-Dependent Diabetes Mellitus of Obese Rats: Impairment in Adipocyte-(Beta-Cell Relationships). Proc. Natl. Acad. Sci. USA.

[23] Tan C.Y., Vidal-Puig A. (2008). Adipose Tissue Expandability: The Metabolic Problems of Obesity May Arise from the Inability to Become More Obese. Biochem. Soc. Trans..

[24] Carobbio S., Rodriguez-Cuenca S., Vidal-Puig A. (2011). Origins of Metabolic Complications in Obesity: Ectopic Fat Accumulation. the Importance of the Qualitative Aspect of Lipotoxicity. Curr. Opin. Clin. Nutr. Metab. Care.

[25] Shi H., Kokoeva M.V., Inouye K., Tzameli I., Yin H., Flier J.S. (2006). TLR4 Links Innate Immunity and Fatty Acid-Induced Insulin Resistance. J. Clin. Investig..

[26] Sajan M.P., Nimal S., Mastorides S., Acevedo-Duncan M., Kahn C.R., Fields A.P., Braun U., Leitges M., Farese R.V. (2012). Correction of Metabolic Abnormalities in a Rodent Model of Obesity, Metabolic Syndrome, and Type 2 Diabetes Mellitus by Inhibitors of Hepatic Protein Kinase C-ι. Metabolism.

[27] Lu X., Bean J.S., Kassab G.S., Rekhter M.D. (2011). Protein Kinase C Inhibition Ameliorates Functional Endothelial Insulin Resistance and Vascular Smooth Muscle Cell Hypersensitivity to Insulin in Diabetic Hypertensive Rats. Cardiovasc. Diabetol..

[28] Jaeschke A., Davis R.J. (2007). Metabolic Stress Signaling Mediated by Mixed-Lineage Kinases. Mol. Cell.

[29] Law B.A., Liao X., Moore K.S., Southard A., Roddy P., Ji R., Szulc Z., Bielawska A., Schulze P.C., Cowart L.A. (2018). Lipotoxic Very-Long-Chain Ceramides Cause Mitochondrial Dysfunction, Oxidative Stress, and Cell Death in Cardiomyocytes. FASEB J..

[30] Yu J., Novgorodov S.A., Chudakova D., Zhu H., Bielawska A., Bielawski J., Obeid L.M., Kindy M.S., Gudz T.I. (2007). JNK3 Signaling Pathway Activates Ceramide Synthase Leading to Mitochondrial Dysfunction. J. Biol. Chem..

[31] Meyer J.G., Softic S., Basisty N., Rardin M.J., Verdin E., Gibson B.W., Ilkayeva O., Newgard C.B., Ronald Kahn C., Schilling B. (2018). Temporal Dynamics of Liver Mitochondrial Protein Acetylation and Succinylation and Metabolites Due to High Fat Diet and/or Excess Glucose or Fructose. PLoS ONE.

[32] Jang H., Park K. (2020). Omega-3 and Omega-6 Polyunsaturated Fatty Acids and Metabolic Syndrome: A Systematic Review and Meta-Analysis. Clin. Nutr..

[33] Lankinen M., Schwab U., Erkkilä A., Seppänen-Laakso T., Hannila M.L., Mussalo H., Lehto S., Uusitupa M., Gylling H., Orešič M. (2009). Fatty Fish Intake Decreases Lipids Related to Inflammation and Insulin Signal–ng—A Lipidomics Approach. PLoS ONE.

[34] Sage A.T., Holtby-Ottenhof S., Shi Y., Damjanovic S., Sharma A.M., Werstuck G.H. (2012). Metabolic Syndrome and Acute Hyperglycemia Are Associated with Endoplasmic Reticulum Stress in Human Mononuclear Cells. Obesity.

[35] Tominaga H., Katoh H., Odagiri K., Takeuchi Y., Kawashima H., Saotome M., Urushida T., Satoh H., Hayashi H. (2008). Different Effects of Palmitoyl-L-Carnitine and Palmitoyl-CoA on Mitochondrial Function in Rat Ventricular Myocytes. Am. J. Physiol. Heart Circ. Physiol..

[36] Holland W.L., Summers S.A. (2008). Sphingolipids, Insulin Resistance, and Metabolic Disease: New Insights from in Vivo Manipulation of Sphingolipid Metabolism. Endocr. Rev..

[37] Siskind L.J., Mullen T.D., Rosales K.R., Clarke C.J., Hernandez-Corbacho M.J., Edinger A.L., Obeid L.M. (2010). The BCL-2 Protein BAK Is Required for Long-Chain Ceramide Generation during Apoptosis. J. Biol. Chem..

[38] Parihar A., Parihar M.S., Nazarewicz R., Ghafourifar P. (2010). Importance of Cytochrome c Redox State for Ceramide-Induced Apoptosis of Human Mammary Adenocarcinoma Cells. Biochim. Biophys. Acta Gen. Subj..

[39] Henique C., Mansouri A., Fumey G., Lenoir V., Girard J., Bouillaud F., Prip-Buus C., Cohen I. (2010). Increased Mitochondrial Fatty Acid Oxidation Is Sufficient to Protect Skeletal Muscle Cells from Palmitate-Induced Apoptosis. J. Biol. Chem..

[40] Raichur S., Wang S.T., Chan P.W., Li Y., Ching J., Chaurasia B., Dogra S., Öhman M.K., Takeda K., Sugii S. (2014). CerS_2_ Haploinsufficiency Inhibits β-Oxidation and Confers Susceptibility to Diet-Induced Steatohepatitis and Insulin Resistance. Cell Metab..

[41] Neeland I.J., Singh S., McGuire D.K., Vega G.L., Roddy T., Reilly D.F., Castro-Perez J., Kozlitina J., Scherer P.E. (2018). Relation of Plasma Ceramides to Visceral Adiposity, Insulin Resistance and the Development of Type 2 Diabetes Mellitus: The Dallas Heart Study. Diabetologia.

[42] Wang D.D., Toledo E., Hruby A., Rosner B.A., Willett W.C., Sun Q., Razquin C., Zheng Y., Ruiz-Canela M., Guasch-Ferré M. (2017). Plasma Ceramides, Mediterranean Diet, and Incident Cardiovascular Disease in the PREDIMED Trial (Prevención Con Dieta Mediterránea). Circulation.

[43] Fretts A.M., Jensen P.N., Hoofnagle A.N., McKnight B., Howard B.V., Umans J., Sitlani C.M., Siscovick D.S., King I.B., Djousse L. (2021). Plasma Ceramides Containing Saturated Fatty Acids Are Associated with Risk of Type 2 Diabetes. J. Lipid Res..

[44] Haus J.M., Kashyap S.R., Kasumov T., Zhang R., Kelly K.R., Defronzo R.A., Kirwan J.P. (2009). Plasma Ceramides Are Elevated in Obese Subjects with Type 2 Diabetes and Correlate with the Severity of Insulin Resistance. Diabetes.

[45] Poss A.M., Krick B., Maschek J.A., Haaland B., Cox J.E., Karra P., Ibele A.R., Hunt S.C., Adams T.D., Holland W.L. (2022). Following Roux-En-Y Gastric Bypass Surgery, Serum Ceramides Demarcate Patients That Will Fail to Achieve Normoglycemia and Diabetes Remission. Med.

[46] Boon J., Hoy A.J., Stark R., Brown R.D., Meex R.C., Henstridge D.C., Schenk S., Meikle P.J., Horowitz J.F., Kingwell B.A. (2013). Ceramides Contained in LDL Are Elevated in Type 2 Diabetes and Promote Inflammation and Skeletal Muscle Insulin Resistance. Diabetes.

[47] Broskey N.T., Obanda D.N., Burton J.H., Cefalu W.T., Ravussin E. (2018). Skeletal Muscle Ceramides and Daily Fat Oxidation in Obesity and Diabetes. Metabolism.

[48] Kelley D.E., Simoneau J.A. (1994). Impaired Free Fatty Acid Utilization by Skeletal Muscle in Non-Insulin-Dependent Diabetes Mellitus. J. Clin. Investig..

[49] Colberg S.R., Simoneau J.A., Thaete F.L., Kelley D.E. (1995). Skeletal Muscle Utilization of Free Fatty Acids in Women with Visceral Obesity. J. Clin. Investig..

[50] Voisin S., Almén M.S., Moschonis G., Chrousos G.P., Manios Y., Schiöth H.B. (2015). Dietary fat quality impacts genome-wide DNA methylation patterns in a cross-sectional study of Greek preadolescents. Eur. J. Hum. Genet..

[51] Khalyfa A., Carreras A., Hakim F., Cunningham J.M., Wang Y., Gozal D. (2013). Effects of late gestational high-fat diet on body weight, metabolic regulation and adipokine expression in offspring. Int. J. Obes..

[52] Barrès R., Osler M.E., Yan J., Rune A., Fritz T., Caidahl K., Krook A., Zierath J.R. (2009). Non-CpG methylation of the PGC-1alpha promoter through DNMT3B controls mitochondrial density. Cell Metab..

[53] Sookoian S., Rosselli M.S., Gemma C., Burgueño A.L., Fernández Gianotti T., Castaño G.O., Pirola C.J. (2010). Epigenetic regulation of insulin resistance in nonalcoholic fatty liver disease: Impact of liver methylation of the peroxisome proliferator-activated receptor γ coactivator 1α promoter. Hepatology.

[54] Zheng L.D., Linarelli L.E., Liu L., Wall S.S., Greenawald M.H., Seidel R.W., Estabrooks P.A., Almeida F.A., Cheng Z. (2015). Insulin resistance is associated with epigenetic and genetic regulation of mitochondrial DNA in obese humans. Clin. Epigenetics.

[55] Pirola C.J., Gianotti T.F., Burgueño A.L., Rey-Funes M., Loidl C.F., Mallardi P., Martino J.S., Castaño G.O., Sookoian S. (2013). Epigenetic modification of liver mitochondrial DNA is associated with histological severity of nonalcoholic fatty liver disease. Gut.

[56] Malik A.N., Simões I.C.M., Rosa H.S., Khan S., Karkucinska-Wieckowska A., Wieckowski M.R. (2019). A Diet Induced Maladaptive Increase in Hepatic Mitochondrial DNA Precedes OXPHOS Defects and May Contribute to Non-Alcoholic Fatty Liver Disease. Cells.

[57] Lee K., Haddad A., Osme A., Kim C., Borzou A., Ilchenko S., Allende D., Dasarathy S., McCullough A., Sadygov R.G. (2018). Hepatic Mitochondrial Defects in a Nonalcoholic Fatty Liver Disease Mouse Model Are Associated with Increased Degradation of Oxidative Phosphorylation Subunits. Mol. Cell. Proteom..

[58] Mihalik S.J., Goodpaster B.H., Kelley D.E., Chace D.H., Vockley J., Toledo F.G.S., Delany J.P. (2010). Increased Levels of Plasma Acylcarnitines in Obesity and Type 2 Diabetes and Identification of a Marker of Glucolipotoxicity. Obesity.

[59] Adams S.H., Hoppel C.L., Lok K.H., Zhao L., Wong S.W., Minkler P.E., Hwang D.H., Newman J.W., Garvey W.T. (2009). Plasma Acylcarnitine Profiles Suggest Incomplete Long-Chain Fatty Acid β-Oxidation and Altered Tricarboxylic Acid Cycle Activity in Type 2 Diabetic African-American Women. J. Nutr..

[60] Nikniaz L., Mahmudiono T., Jasim S.A., Vajdi M., Thangavelu L., Farhangi M.A. (2022). Nutrient Pattern Analysis of Mineral Based, Simple Sugar Based, and Fat Based Diets and Risk of Metabolic Syndrome: A Comparative Nutrient Panel. BMC Endocr. Disord..

[61] Yamazaki M., Munetsuna E., Yamada H., Ando Y., Mizuno G., Murase Y., Kondo K., Ishikawa H., Teradaira R., Suzuki K. (2016). Fructose Consumption Induces Hypomethylation of Hepatic Mitochondrial DNA in Rats. Life Sci..

[62] Lanaspa M.A., Sanchez-Lozada L.G., Choi Y.J., Cicerchi C., Kanbay M., Roncal-Jimenez C.A., Ishimoto T., Li N., Marek G., Duranay M. (2012). Uric Acid Induces Hepatic Steatosis by Generation of Mitochondrial Oxidative Stress: Potential Role in Fructose-Dependent and -Independent Fatty Liver. J. Biol. Chem..

[63] Jaiswal N., Maurya C.K., Arha D., Avisetti D.R., Prathapan A., Raj P.S., Raghu K.G., Kalivendi S.V., Tamrakar A.K. (2015). Fructose Induces Mitochondrial Dysfunction and Triggers Apoptosis in Skeletal Muscle Cells by Provoking Oxidative Stress. Apoptosis.

[64] Rodríguez L.A., Madsen K.A., Cotterman C., Lustig R.H. (2016). Added Sugar Intake and Metabolic Syndrome in US Adolescents: Cross-Sectional Analysis of the National Health and Nutrition Examination Survey 2005–2012. Public Health Nutr..

[65] Chiavaroli L., de Souza R.J., Ha V., Cozma A.I., Mirrahimi A., Wang D.D., Yu M., Carleton A.J., di Buono M., Jenkins A.L. (2015). Effect of Fructose on Established Lipid Targets: A Systematic Review and Meta-Analysis of Controlled Feeding Trials. J. Am. Heart Assoc..

[66] Seidelmann S.B., Claggett B., Cheng S., Henglin M., Shah A., Steffen L.M., Folsom A.R., Rimm E.B., Willett W.C., Solomon S.D. (2018). Dietary Carbohydrate Intake and Mortality: A Prospective Cohort Study and Meta-Analysis. Lancet Public Health.

[67] Semnani-Azad Z., Khan T.A., Blanco Mejia S., de Souza R.J., Leiter L.A., Kendall C.W.C., Hanley A.J., Sievenpiper J.L. (2020). Association of Major Food Sources of Fructose-Containing Sugars with Incident Metabolic Syndrome: A Systematic Review and Meta-Analysis. JAMA Netw. Open.

[68] Walker M.E., Xanthakis V., Moore L.L., Vasan R.S., Jacques P.F. (2020). Cumulative Sugar-Sweetened Beverage Consumption Is Associated with Higher Concentrations of Circulating Ceramides in the Framingham Offspring Cohort. Am. J. Clin. Nutr..

[69] Malik V.S., Popkin B.M., Bray G.A., Després J.P., Willett W.C., Hu F.B. (2010). Sugar-Sweetened Beverages and Risk of Metabolic Syndrome and Type 2 Diabetes: A Meta-Analysis. Diabetes Care.

[70] Zhang X., Li X., Liu L., Hong F., Zhao H., Chen L., Zhang J., Jiang Y., Zhang J., Luo P. (2021). Dose-Response Association between Sugar- And Artificially Sweetened Beverage Consumption and the Risk of Metabolic Syndrome: A Meta-Analysis of Population-Based Epidemiological Studies. Public Health Nutr..

[71] Park S., Ahn J., Kim N.S., Lee B.K. (2017). High Carbohydrate Diets Are Positively Associated with the Risk of Metabolic Syndrome Irrespective to Fatty Acid Composition in Women: The KNHANES 2007–2014. Int. J. Food Sci. Nutr..

[72] Chan A.M.L., Ng A.M.H., Mohd Yunus M.H., Idrus R.B.H., Law J.X., Yazid M.D., Chin K.Y., Shamsuddin S.A., Lokanathan Y. (2021). Recent Developments in Rodent Models of High-Fructose Diet-Induced Metabolic Syndrome: A Systematic Review. Nutrients.

[73] Stubbs C.D., Smith A.D. (1984). The modification of mammalian membrane polyunsaturated fatty acid composition in relation to membrane fluidity and function. Biochim. Et Biophys. Acta..

[74] Calder P.C. (2002). Dietary Modification of Inflammation with Lipids. Proc. Nutr. Soc..

[75] White P.J., Arita M., Taguchi R., Kang J.X., Marette A. (2010). Transgenic Restoration of Long-Chain n-3 Fatty Acids in Insulin Target Tissues Improves Resolution Capacity and Alleviates Obesity-Linked Inflammation and Insulin Resistance in High-Fat-Fed Mice. Diabetes.

[76] Dong Y.Q., Zhang X.Z., Sun L.L., Zhang S.Y., Liu B., Liu H.Y., Wang X., Jiang C.T. (2017). Omega-3 PUFA Ameliorates Hyperhomocysteinemia-Induced Hepatic Steatosis in Mice by Inhibiting Hepatic Ceramide Synthesis. Acta Pharm. Sin..

[77] Taltavull N., Ras R., Mariné S., Romeu M., Giralt M., Méndez L., Medina I., Ramos-Romero S., Torres J.L., Nogués M.R. (2016). Protective Effects of Fish Oil on Pre-Diabetes: A Lipidomic Analysis of Liver Ceramides in Rats. Food Funct..

[78] Camacho-Muñoz D., Niven J., Kucuk S., Cucchi D., Certo M., Jones S.W., Fischer D.P., Mauro C., Nicolaou A. (2022). Omega-3 Polyunsaturated Fatty Acids Reverse the Impact of Western Diets on Regulatory T Cell Responses through Averting Ceramide-Mediated Pathways. Biochem. Pharm..

[79] Pinel A., Rigaudière J.P., Laillet B., Pouyet C., Malpuech-Brugère C., Prip-Buus C., Morio B., Capel F. (2016). N-3PUFA Differentially Modulate Palmitate-Induced Lipotoxicity through Alterations of Its Metabolism in C2C12 Muscle Cells. Biochim. Biophys. Acta Mol. Cell Biol. Lipids.

[80] Khalili L., Valdes-Ramos R., Harbige L.S. (2021). Effect of N-3 (Omega-3) Polyunsaturated Fatty Acid Supplementation on Metabolic and Inflammatory Biomarkers and Body Weight in Patients with Type 2 Diabetes Mellitus: A Systematic Review and Meta-Analysis of RCTs. Metabolites.

[81] O’Mahoney L.L., Matu J., Price O.J., Birch K.M., Ajjan R.A., Farrar D., Tapp R., West D.J., Deighton K., Campbell M.D. (2018). Omega-3 Polyunsaturated Fatty Acids Favourably Modulate Cardiometabolic Biomarkers in Type 2 Diabetes: A Meta-Analysis and Meta-Regression of Randomized Controlled Trials. Cardiovasc. Diabetol..

[82] Li N., Yue H., Jia M., Liu W., Qiu B., Hou H., Huang F., Xu T. (2019). Effect of Low-Ratio n-6/n-3 PUFA on Blood Glucose: A Meta-Analysis. Food Funct..

[83] Liu R., Chen L., Wang Y., Zhang G., Cheng Y., Feng Z., Bai X., Liu J. (2020). High Ratio of ω-3/ω-6 Polyunsaturated Fatty Acids Targets MTORC1 to Prevent High-Fat Diet-Induced Metabolic Syndrome and Mitochondrial Dysfunction in Mice. J. Nutr. Biochem..

[84] Zhang Y., Jiang L., Hu W., Zheng Q., Xiang W. (2011). Mitochondrial Dysfunction during in Vitro Hepatocyte Steatosis Is Reversed by Omega-3 Fatty Acid-Induced up-Regulation of Mitofusin 2. Metabolism.

[85] Golpour P., Nourbakhsh M., Mazaherioun M., Janani L., Nourbakhsh M., Yaghmaei P. (2020). Improvement of NRF2 Gene Expression and Antioxidant Status in Patients with Type 2 Diabetes Mellitus after Supplementation with Omega-3 Polyunsaturated Fatty Acids: A Double-Blind Randomised Placebo-Controlled Clinical Trial. Diabetes Res. Clin. Pr..

[86] Mori T.A., Puddey I.B., Burke V., Croft K.D., Dunstan D.W., Rivera J.H., Beilin L.J. (2000). Effect of Ω3 Fatty Acids on Oxidative Stress in Humans: GC-MS Measurement of Urinary F2-Isoprostane Excretion. Redox Rep..

[87] Heshmati J., Morvaridzadeh M., Maroufizadeh S., Akbari A., Yavari M., Amirinejad A., Maleki-Hajiagha A., Sepidarkish M. (2019). Omega-3 Fatty Acids Supplementation and Oxidative Stress Parameters: A Systematic Review and Meta-Analysis of Clinical Trials. Pharmacol. Res..

[88] Okada L.S.D.R.R., Oliveira C.P., Stefano J.T., Nogueira M.A., da Silva I.D.C.G., Cordeiro F.B., Alves V.A.F., Torrinhas R.S., Carrilho F.J., Puri P. (2018). Omega-3 PUFA Modulate Lipogenesis, ER Stress, and Mitochondrial Dysfunction Markers in NASH—Proteomic and Lipidomic Insight. Clin. Nutr..

[89] Coll T., Eyre E., Rodríguez-Calvo R., Palomer X., Sánchez R.M., Merlos M., Laguna J.C., Vázquez-Carrera M. (2008). Oleate Reverses Palmitate-Induced Insulin Resistance and Inflammation in Skeletal Muscle Cells. J. Biol. Chem..

[90] Barbier O., Torra I.P., Duguay Y., Blanquart C., Fruchart J.C., Glineur C., Staels B. (2002). Pleiotropic Actions of Peroxisome Proliferator-Activated Receptors in Lipid Metabolism and Atherosclerosis. Arter. Thromb. Vasc. Biol..

[91] London E., Bloyd M., Stratakis C.A. (2020). PKA Functions in Metabolism and Resistance to Obesity: Lessons from Mouse and Human Studies. J. Endocrinol..

[92] Liu X., Kris-Etherton P.M., West S.G., Lamarche B., Jenkins D.J.A., Fleming J.A., McCrea C.E., Pu S., Couture P., Connelly P.W. (2016). Effects of Canola and High-Oleic-Acid Canola Oils on Abdominal Fat Mass in Individuals with Central Obesity. Obesity.

[93] Lasa A., Miranda J., Bulló M., Casas R., Salas-Salvadó J., Larretxi I., Estruch R., Ruiz-Gutiérrez V., Portillo M.P. (2014). Comparative Effect of Two Mediterranean Diets versus a Low-Fat Diet on Glycaemic Control in Individuals with Type 2 Diabetes. Eur. J. Clin. Nutr..

[94] Sureda A., Bibiloni M.D., Martorell M., Buil-Cosiales P., Marti A., Pons A., Tur J.A., Martinez-Gonzalez M.Á. (2016). Mediterranean Diets Supplemented with Virgin Olive Oil and Nuts Enhance Plasmatic Antioxidant Capabilities and Decrease Xanthine Oxidase Activity in People with Metabolic Syndrome: The PREDIMED Study. Mol. Nutr. Food Res..

[95] Venturini D., Simão A.N.C., Urbano M.R., Dichi I. (2015). Effects of Extra Virgin Olive Oil and Fish Oil on Lipid Profile and Oxidative Stress in Patients with Metabolic Syndrome. Nutrition.

[96] Pastor R., Bouzas C., Tur J.A. (2021). Beneficial Effects of Dietary Supplementation with Olive Oil, Oleic Acid, or Hydroxytyrosol in Metabolic Syndrome: Systematic Review and Meta-Analysis. Free Radic. Biol. Med..

[97] Patti A.M., Carruba G., Cicero A.F.G., Banach M., Nikolic D., Giglio R.V., Terranova A., Soresi M., Giannitrapani L., Montalto G. (2020). Daily Use of Extra Virgin Olive Oil with High Oleocanthal Concentration Reduced Body Weight, Waist Circumference, Alanine Transaminase, Inflammatory Cytokines and Hepatic Steatosis in Subjects with the Metabolic Syndrome: A 2-Month Intervention Study. Metabolites.

[98] Salas-Salvadó J., Bulló M., Babio N., Martínez-González M.Á., Ibarrola-Jurado N., Basora J., Estruch R., Covas M.I., Corella D., Arós F. (2011). Reduction in the Incidence of Type 2 Diabetes with the Mediterranean Diet: Results of the PREDIMED-Reus Nutrition Intervention Randomized Trial. Diabetes Care.

[99] Schwingshackl L., Lampousi A.M., Portillo M.P., Romaguera D., Hoffmann G., Boeing H. (2017). Olive Oil in the Prevention and Management of Type 2 Diabetes Mellitus: A Systematic Review and Meta-Analysis of Cohort Studies and Intervention Trials. Nutr Diabetes.

[100] DeLany J.P., Windhauser M.M., Champagne C.M., Bray G.A. (2000). Differential Oxidation of Individual Dietary Fatty Acids in Humans. Am. J. Clin. Nutr..

[101] Schönfeld P., Wojtczak L. (2016). Short- and Medium-Chain Fatty Acids in Energy Metabolism: The Cellular Perspective. J. Lipid Res..

[102] Tham Y.Y., Choo Q.C., Muhammad T.S.T., Chew C.H. (2020). Lauric Acid Alleviates Insulin Resistance by Improving Mitochondrial Biogenesis in THP-1 Macrophages. Mol. Biol. Rep..

[103] Portillo M., Serra F., Simon E., del Barrio A., Palou A. (1998). Energy Restriction with High-Fat Diet Enriched with Coconut Oil Gives Higher UCP1 and Lower White Fat in Rats. Int. J. Obes..

[104] Schulz N., Himmelbauer H., Rath M., van Weeghel M., Houten S., Kulik W., Suhre K., Scherneck S., Vogel H., Kluge R. (2011). Role of Medium- and Short-Chain L-3-Hydroxyacyl- CoA Dehydrogenase in the Regulation of Body Weight and Thermogenesis. Endocrinology.

[105] Scalfi L., Coltorti A., Contaldo F. (1991). Postprandial Thermogenesis in Lean and Obese Subjects after Meals Supplemented with Medium-Chain and Long-Chain triglycerides. Am. J. Clin. Nutr..

[106] St-Onge M.-P., Ross R., Parsons W.D., Jones P.J.H. (2003). Medium-Chain Triglycerides Increase Energy Expenditure and Decrease Adiposity in Overweight Men. Obes. Res..

[107] Mumme K., Stonehouse W. (2015). Effects of Medium-Chain Triglycerides on Weight Loss and Body Composition: A Meta-Analysis of Randomized Controlled Trials. J. Acad. Nutr. Diet..

[108] Middleton P., Vergis N. (2021). Mitochondrial Dysfunction and Liver Disease: Role, Relevance, and Potential for Therapeutic Modulation. Therap. Adv. Gastroenterol..

[109] Ronis M.J.J., Baumgardner J.N., Sharma N., Vantrease J., Ferguson M., Tong Y., Wu X., Cleves M.A., Badger T.M. (2013). Medium Chain Triglycerides Dose-Dependently Prevent Liver Pathology in a Rat Model of Non-Alcoholic Fatty Liver Disease. Exp. Biol. Med..

[110] Ströher D.J., de Oliveira M.F., Martinez-Oliveira P., Pilar B.C., Cattelan M.D.P., Rodrigues E., Bertolin K., Gonçalves P.B.D., Piccoli J.D.C.E., Manfredini V. (2020). Virgin Coconut Oil Associated with High-Fat Diet Induces Metabolic Dysfunctions, Adipose Inflammation, and Hepatic Lipid Accumulation. J. Med. Food.

[111] Panth N., Abbott K.A., Dias C.B., Wynne K., Garg M.L. (2018). Differential Effects of Medium- and Long-Chain Saturated Fatty Acids on Blood Lipid Profile: A Systematic Review and Meta-Analysis. Am. J. Clin. Nutr..

[112] Teng M., Zhao Y.J., Khoo A.L., Yeo T.C., Yong Q.W., Lim B.P. (2020). Impact of Coconut Oil Consumption on Cardiovascular Health: A Systematic Review and Meta-Analysis. Nutr. Rev..

[113] Ghadir M.R., Riahin A.A., Havaspour A., Nooranipour M., Habibinejad A.A. (2010). The Relationship between Lipid Profile and Severity of Liver Damage in Cirrhotic Patients. Hepat. Mon..

[114] Adeyemi W.J., Olayaki L.A., Abdussalam T.A., Toriola A.P., Olowu A.B., Yakub A.J., Raji A.O. (2020). Investigation of the Effects of Dietary Modification in Experimental Obesity: Low Dose of Virgin Coconut Oil Has a Potent Therapeutic Value. Biomed. Pharmacother..

[115] Narayanankutty A., Mukesh R.K., Ayoob S.K., Ramavarma S.K., Suseela I.M., Manalil J.J., Kuzhivelil B.T., Raghavamenon A.C. (2016). Virgin Coconut Oil Maintains Redox Status and Improves Glycemic Conditions in High Fructose Fed Rats. J. Food Sci. Technol..

[116] Narayanankutty A., Palliyil D.M., Kuruvilla K., Raghavamenon A.C. (2018). Virgin Coconut Oil Reverses Hepatic Steatosis by Restoring Redox Homeostasis and Lipid Metabolism in Male Wistar Rats. J. Sci. Food Agric..

[117] Miyagawa Y., Mori T., Goto K., Kawahara I., Fujiwara-Tani R., Kishi S., Sasaki T., Fujii K., Ohmori H., Kuniyasu H. (2018). Intake of medium-chain fatty acids induces myocardial oxidative stress and atrophy. Lipids Health Dis..

[118] Nikooei P., Hosseinzadeh-Attar M.J., Asghari S., Norouzy A., Yaseri M., Vasheghani-Farahani A. (2021). Effects of Virgin Coconut Oil Consumption on Metabolic Syndrome Components and Asymmetric Dimethylarginine: A Randomized Controlled Clinical Trial. Nutr. Metab. Cardiovasc. Dis..

[119] St-Onge M.-P., Bosarge A., Lee Goree L.T., Darnell B., Hospital R., York N. (2008). Medium Chain Triglyceride Oil Consumption as Part of a Weight Loss Diet Does Not Lead to an Adverse Metabolic Profile When Compared to Olive Oil. J. Am. Coll. Nutr..

[120] de Oliveira Chamma C.M., Bargut T.C.L., Mandarim-De-Lacerda C.A., Aguila M.B. (2017). A Rich Medium-Chain Triacylglycerol Diet Benefits Adiposity but Has Adverse Effects on the Markers of Hepatic Lipogenesis and Beta-Oxidation. Food Funct..

[121] Yamaguchi N., Mahbub M.H., Takahashi H., Hase R., Ishimaru Y., Sunagawa H., Amano H., Kobayashi- Miura M., Kanda H., Fujita Y. (2017). Plasma Free Amino Acid Profiles Evaluate Risk of Metabolic Syndrome, Diabetes, Dyslipidemia, and Hypertension in a Large Asian Population. Env. Health Prev. Med..

[122] Ntzouvani A., Nomikos T., Panagiotakos D., Fragopoulou E., Pitsavos C., McCann A., Ueland P.M., Antonopoulou S. (2017). Amino Acid Profile and Metabolic Syndrome in a Male Mediterranean Population: A Cross-Sectional Study. Nutr. Metab. Cardiovasc. Dis..

[123] Weng L., Quinlivan E., Gong Y., Beitelshees A.L., Shahin M.H., Turner S.T., Chapman A.B., Gums J.G., Johnson J.A., Frye R.F. (2015). Association of Branched and Aromatic Amino Acids Levels with Metabolic Syndrome and Impaired Fasting Glucose in Hypertensive Patients. Metab. Syndr. Relat. Disord..

[124] Yamakado M., Nagao K., Imaizumi A., Tani M., Toda A., Tanaka T., Jinzu H., Miyano H., Yamamoto H., Daimon T. (2015). Plasma Free Amino Acid Profiles Predict Four-Year Risk of Developing Diabetes, Metabolic Syndrome, Dyslipidemia, and Hypertension in Japanese Population. Sci. Rep..

[125] Cheng S., Wiklund P., Autio R., Borra R., Ojanen X., Xu L., Törmäkangas T., Alen M. (2015). Adipose Tissue Dysfunction and Altered Systemic Amino Acid Metabolism Are Associated with Non-Alcoholic Fatty Liver Disease. PLoS ONE.

[126] Pirola C.J., Garaycoechea M., Flichman D., Castaño G.O., Sookoian S. (2021). Liver mitochondrial DNA damage and genetic variability of Cytochrome b—A key component of the respirasome—drive the severity of fatty liver disease. J. Intern. Med..

[127] Sunny N.E., Parks E.J., Browning J.D., Burgess S.C. (2011). Excessive hepatic mitochondrial TCA cycle and gluconeogenesis in humans with nonalcoholic fatty liver disease. Cell Metab..

[128] Satapati S., Sunny N.E., Kucejova B., Fu X., He T.T., Méndez-Lucas A., Shelton J.M., Perales J.C., Browning J.D., Burgess S.C. (2012). Elevated TCA cycle function in the pathology of diet-induced hepatic insulin resistance and fatty liver. J. Lipid Res..

[129] Sookoian S., Castaño G.O., Scian R., Fernández Gianotti T., Dopazo H., Rohr C., Gaj G., San Martino J., Sevic I., Flichman D. (2016). Serum aminotransferases in nonalcoholic fatty liver disease are a signature of liver metabolic perturbations at the amino acid and Krebs cycle level. Am. J. Clin. Nutr..

[130] Libert D.M., Nowacki A.S., Natowicz M.R. (2018). Metabolomic Analysis of Obesity, Metabolic Syndrome, and Type 2 Diabetes: Amino Acid and Acylcarnitine Levels Change along a Spectrum of Metabolic Wellness. PeerJ.

[131] Hoppel C.L., Genuth S.M. (1980). Carnitine metabolism in normal-weight and obese human subjects during fasting. Am. J. Physiol..

[132] Schooneman M.G., Vaz F.M., Houten S.M., Soeters M.R. (2013). Acylcarnitines: Reflecting or inflicting insulin resistance?. Diabetes.

[133] Rousseau M., Guénard F., Garneau V., Allam-Ndoul B., Lemieux S., Pérusse L., Vohl M.C. (2019). Associations Between Dietary Protein Sources, Plasma BCAA and Short-Chain Acylcarnitine Levels in Adults. Nutrients.

[134] Gong L.L., Yang S., Zhang W., Han F.F., Xuan L.L., Lv Y.L., Liu H., Liu L.H., Tu W.J. (2020). Targeted Metabolomics for Plasma Amino Acids and Carnitines in Patients with Metabolic Syndrome Using HPLC-MS/MS. Dis Markers.

[135] Yin X., Subramanian S., Willinger C.M., Chen G., Juhasz P., Courchesne P., Chen B.H., Li X., Hwang S.J., Fox C.S. (2016). Metabolite Signatures of Metabolic Risk Factors and Their Longitudinal Changes. J. Clin. Endocrinol. Metab..

[136] Tochikubo O., Nakamura H., Jinzu H., Nagao K., Yoshida H., Kageyama N., Miyano H. (2016). Weight Loss Is Associated with Plasma Free Amino Acid Alterations in Subjects with Metabolic Syndrome. Nutr Diabetes.

[137] Kamaura M., Nishijima K., Takahashi M., Ando T., Mizushima S., Tochikubo O. (2010). Lifestyle Modification in Metabolic Syndrome and Associated Changes in Plasma Amino Acid Profiles. Circ. J..

[138] Hosseinpour-Niazi S., Tahmasebinejad Z., Esfandiar Z., Bakhshi B., Mirmiran P., Azizi F. (2020). Weight Gain, but Not Macronutrient Intake, Modifies the Effect of Dietary Branch Chain Amino Acids on the Risk of Metabolic Syndrome. Diabetes Res. Clin. Pract..

[139] Badely M., Sepandi M., Samadi M., Parastouei K., Taghdir M. (2019). The Effect of Whey Protein on the Components of Metabolic Syndrome in Overweight and Obese Individuals; a Systematic Review and Meta-Analysis. Diabetes Metab. Syndr. Clin. Res. Rev..

[140] Amirani E., Milajerdi A., Reiner Ž., Mirzaei H., Mansournia M.A., Asemi Z. (2020). Effects of Whey Protein on Glycemic Control and Serum Lipoproteins in Patients with Metabolic Syndrome and Related Conditions: A Systematic Review and Meta-Analysis of Randomized Controlled Clinical Trials. Lipids Health Dis..

[141] Chalvon-Demersay T., Azzout-Marniche D., Arfsten J., Egli L., Gaudichon C., Karagounis L.G., Tomé D. (2017). A Systematic Review of the Effects of Plant Compared with Animal Protein Sources on Features of Metabolic Syndrome1-3. J. Nutr..

[142] Ostman C., Smart N.A., Morcos D., Duller A., Ridley W., Jewiss D. (2017). The Effect of Exercise Training on Clinical Outcomes in Patients with the Metabolic Syndrome: A Systematic Review and Meta-Analysis. Cardiovasc. Diabetol..

[143] Powers S.K., Deminice R., Ozdemir M., Yoshihara T., Bomkamp M.P., Hyatt H. (2020). Exercise-Induced Oxidative Stress: Friend or Foe?. J. Sport Health Sci..

[144] Heo J.W., No M.H., Cho J., Choi Y., Cho E.J., Park D.H., Kim T.W., Kim C.J., Seo D.Y., Han J. (2021). Moderate Aerobic Exercise Training Ameliorates Impairment of Mitochondrial Function and Dynamics in Skeletal Muscle of High-Fat Diet-Induced Obese Mice. FASEB J..

[145] Silva L.A., Pinho C.A., Scarabelot K.S., Fraga D.B., Volpato A.M.J., Boeck C.R., Souza C.T., Streck E.L., Pinho R.A. (2009). Physical Exercise Increases Mitochondrial Function and Reduces Oxidative Damage in Skeletal Muscle. Eur. J. Appl. Physiol..

[146] Liang X., Liu L., Fu T., Zhou Q., Zhou D., Xiao L., Liu J., Kong Y., Xie H., Yi F. (2016). Exercise Inducible Lactate Dehydrogenase B Regulates Mitochondrial Function in Skeletal Muscle. J. Biol. Chem..

[147] Yakeu G., Butcher L., Isa S., Webb R., Roberts A.W., Thomas A.W., Backx K., James P.E., Morris K. (2010). Low-intensity exercise enhances expression of markers of alternative activation in circulating leukocytes: Roles of PPARγ and Th2 cytokines. Atherosclerosis.

[148] Butcher L.R., Thomas A., Backx K., Roberts A., Webb R., Morris K. (2008). Low-intensity exercise exerts beneficial effects on plasma lipids via PPARgamma. Med. Sci. Sports Exerc..

[149] Krammer U.D.B., Sommer A., Tschida S., Mayer A., Lilja S.V., Switzeny O.J., Hippe B., Rust P., Haslberger A.G. (2022). *PGC-1α* Methylation, miR-23a, and miR-30e Expression as Biomarkers for Exercise- and Diet-Induced Mitochondrial Biogenesis in Capillary Blood from Healthy Individuals: A Single-Arm Intervention. Sports.

[150] Phielix E., Meex R., Moonen-Kornips E., Hesselink M.K.C., Schrauwen P. (2010). Exercise Training Increases Mitochondrial Content and Ex Vivo Mitochondrial Function Similarly in Patients with Type 2 Diabetes and in Control Individuals. Diabetologia.

[151] Meex R.C.R., Schrauwen-Hinderling V.B., Moonen-Kornips E., Schaart G., Mensink M., Phielix E., van de Weijer T., Sels J.P., Schrauwen P., Hesselink M.K.C. (2010). Restoration of Muscle Mitochondrial Function and Metabolic Flexibility in Type 2 Diabetes by Exercise Training Is Paralleled by Increased Myocellular Fat Storage and Improved Insulin Sensitivity. Diabetes.

[152] Houttu V., Bouts J., Vali Y., Daams J., Grefhorst A., Nieuwdorp M., Holleboom A.G. (2022). Does aerobic exercise reduce NASH and liver fibrosis in patients with non-alcoholic fatty liver disease? A systematic literature review and meta-analysis. Front. Endocrinol..

[153] Warren J.L., Hunter G.R., Gower B.A., Bamman M.M., Windham S.T., Moellering D.R., Fisher G. (2020). Exercise Effects on Mitochondrial Function and Lipid Metabolism during Energy Balance. Med. Sci. Sport. Exerc..

[154] Porter C., Reidy P.T., Bhattarai N., Sidossis L.S., Rasmussen B.B. (2015). Resistance Exercise Training Alters Mitochondrial Function in Human Skeletal Muscle. Med. Sci. Sport. Exerc..

[155] Kasumov T., Solomon T.P.J., Hwang C., Huang H., Haus J.M., Zhang R., Kirwan J.P. (2015). Improved Insulin Sensitivity after Exercise Training Is Linked to Reduced Plasma C14:0 Ceramide in Obesity and Type 2 Diabetes. Obesity.

[156] Dubé J.J., Amati F., Toledo F.G.S., Stefanovic-Racic M., Rossi A., Coen P., Goodpaster B.H. (2011). Effects of Weight Loss and Exercise on Insulin Resistance, and Intramyocellular Triacylglycerol, Diacylglycerol and Ceramide. Diabetologia.

[157] Rivas D.A., Morris E.P., Haran P.H., Pasha E.P., Da M., Morais S., Dolnikowski G.G., Phillips E.M., Fielding R.A. (2012). Increased Ceramide Content and NFB Signaling May Contribute to the Attenuation of Anabolic Signaling after Resistance Exercise in Aged Males. J. Appl. Physiol..

[158] Alizaei Yousefabadi H., Niyazi A., Alaee S., Fathi M., Mohammad Rahimi G.R. (2021). Anti-Inflammatory Effects of Exercise on Metabolic Syndrome Patients: A Systematic Review and Meta-Analysis. Biol. Res. Nurs..

[159] Wewege M.A., Thom J.M., Rye K.A., Parmenter B.J. (2018). Aerobic, Resistance or Combined Training: A Systematic Review and Meta-Analysis of Exercise to Reduce Cardiovascular Risk in Adults with Metabolic Syndrome. Atherosclerosis.

[160] Liang M., Pan Y., Zhong T., Zeng Y., Cheng A.S.K. (2021). Effects of Aerobic, Resistance, and Combined Exercise on Metabolic Syndrome Parameters and Cardiovascular Risk Factors: A Systematic Review and Network Meta-Analysis. Rev. Cardiovasc. Med..

[161] Anderssen S.A., Carroll S., Urdal P., Holme I. (2007). Combined Diet and Exercise Intervention Reverses the Metabolic Syndrome in Middle-Aged Males: Results from the Oslo Diet and Exercise Study. Scand. J. Med. Sci. Sport..

[162] Joseph M.S., Tincopa M.A., Walden P., Jackson E., Conte M.L., Rubenfire M. (2019). The Impact of Structured Exercise Programs on Metabolic Syndrome and Its Components: A Systematic Review. Diabetes Metab. Syndr. Obes..

[163] Kouki R., Schwab U., Lakka T.A., Hassinen M., Savonen K., Komulainen P., Krachler B., Rauramaa R. (2012). Diet, Fitness and Metabolic Syndrome—The DR’s EXTRA Study. Nutr. Metab. Cardiovasc. Dis..

